# PDXNet portal: patient-derived Xenograft model, data, workflow and tool discovery

**DOI:** 10.1093/narcan/zcac014

**Published:** 2022-04-22

**Authors:** Soner Koc, Michael W Lloyd, Jeffrey W Grover, Nan Xiao, Sara Seepo, Sai Lakshmi Subramanian, Manisha Ray, Christian Frech, John DiGiovanna, Phillip Webster, Steven Neuhauser, Anuj Srivastava, Xing Yi Woo, Brian J Sanderson, Brian White, Paul Lott, Lacey E Dobrolecki, Heidi Dowst, PDXNet Consortium, Matthew Bailey, Emilio Cortes-Sanchez, Sandra Scherer, Chieh-Hsiang Yang, Maihi Fujita, Zhengtao Chu, Ling Zhao, Andrew Butterfield, Argun Akcakanat, Gao Boning, Kurt Evans, Bingliang Fang, Don Gibbons, Vanessa Jensen, Dara Keener, Michael Kim, Scott Kopetz, Mourad Majidi, David Menter, John Minna, Hyunsil Park, Fei Yang, Brenda Timmons, Jing Wang, Shannon Westin, Timothy Yap, Jianhua Zhang, Ran Zhang, Min Jin Ha, Huiqin Chen, Yuanxin Xi, Luc Girard, Erkan Yucan, Bryce P Kirby, Bingbing Dai, Yi Xu, Alexey Sorokin, Kelly Gale, Jithesh Augustine, Stephen Scott, Ismail Meraz, Dylan Fingerman, Andrew Kossenkov, Qin Liu, Min Xiao, Jayamanna Wickramasinghe, Haiyin Lin, Eric Ramirez-Salazar, Kate Nathanson, Mike Tetzlaff, George Xu, Vashisht G Yennu-Nanda, Rebecca Aft, Jessica Andrews, Alicia Asaro, Song Cao, Feng Chen, Sherri Davies, John DiPersio, Ryan Fields, Steven Foltz, Katherine Fuh, Kian Lim, Jason Held, Jeremy Hoog, Reyka G Jayasinghe, Yize Li, Jinqin Luo, Cynthia Ma, Jay Mashl, Chia-Kuei Mo, Fernanda Rodriguez, Hua Sun, Nadezhda V Terekhanova, Rose Tipton, Brian VanTine, Andrea Wang-Gillam, Mike Wendl, Yige Wu, Matt Wyczalkowski, Lijun Yao, Daniel Cui Zhou, Matthew Ellis, Michael Ittmann, Susan Hilsenbeck, Bert O’Malley, Amanda Kirane, May Cho, David Gandara, Jonathan Reiss, Tiffany Le, Ralph De Vere White, Cliff Tepper, David Cooke, Luis Godoy, Lisa Brown, Marc Dall’Era, Christopher Evans, Rashmi Verma, Sepideh Gholami, David J Segal, John Albeck, Edward Pugh, Susan Stewart, David Rocke, Hongyong Zhang, Nicole Coggins, Ana Estrada, Ted Toal, Alexa Morales, Guadalupe Polanco Echeverry, Sienna Rocha, Ai-Hong Ma, Yvonne A Evrard, Tiffany A Wallace, Jeffrey A Moscow, James H Doroshow, Nicholas Mitsiades, Salma Kaochar, Chong-xian Pan, Moon S Chen, Luis Carvajal-Carmona, Alana L Welm, Bryan E Welm, Michael T Lewis, Ramaswamy Govindan, Li Ding, Shunqiang Li, Meenhard Herlyn, Michael A Davies, Jack Roth, Funda Meric-Bernstam, Peter N Robinson, Carol J Bult, Brandi Davis-Dusenbery, Dennis A Dean, Jeffrey H Chuang

**Affiliations:** Seven Bridges, Charlestown, MA 02129, USA; The Jackson Laboratory, Bar Harbor, ME 04609, USA; Seven Bridges, Charlestown, MA 02129, USA; Seven Bridges, Charlestown, MA 02129, USA; Seven Bridges, Charlestown, MA 02129, USA; Seven Bridges, Charlestown, MA 02129, USA; Seven Bridges, Charlestown, MA 02129, USA; Seven Bridges, Charlestown, MA 02129, USA; Seven Bridges, Charlestown, MA 02129, USA; Seven Bridges, Charlestown, MA 02129, USA; The Jackson Laboratory, Bar Harbor, ME 04609, USA; The Jackson Laboratory for Genomic Medicine, Farmington, CT 06032, USA; The Jackson Laboratory for Genomic Medicine, Farmington, CT 06032, USA; The Jackson Laboratory for Genomic Medicine, Farmington, CT 06032, USA; The Jackson Laboratory for Genomic Medicine, Farmington, CT 06032, USA; University of California - Davis, Davis, CA 95616, USA; Baylor College of Medicine, Houston, TX 77030, USA; Baylor College of Medicine, Houston, TX 77030, USA; Leidos Biomedical Research, Inc, Frederick National Laboratory for Cancer Research, Frederick, MD 21701, USA; Center to Reduce Health Disparities, National Cancer Institute, Bethesda, MD 20814, USA; Investigational Drug Branch, National Cancer Institute, Bethesda, MD 20814, USA; Division of Cancer Treatment and Diagnosis, National Cancer Institute, Bethesda, MD 20814, USA; Baylor College of Medicine, Houston, TX 77030, USA; Baylor College of Medicine, Houston, TX 77030, USA; University of California - Davis, Davis, CA 95616, USA; Harvard Medical School, West Roxbury, MA 02115, USA; University of California - Davis, Davis, CA 95616, USA; University of California - Davis, Davis, CA 95616, USA; Huntsman Cancer Institute, Salt Lake City, UT 84112, USA; Huntsman Cancer Institute, Salt Lake City, UT 84112, USA; Baylor College of Medicine, Houston, TX 77030, USA; Washington University School of Medicine, St. Louis, MO 63110, USA; Washington University School of Medicine, St. Louis, MO 63110, USA; Washington University School of Medicine, St. Louis, MO 63110, USA; The Wistar Institute, Philadelphia, PA 19104, USA; The University of Texas MD Anderson Cancer Center, Houston, TX 77030, USA; The University of Texas MD Anderson Cancer Center, Houston, TX 77030, USA; The University of Texas MD Anderson Cancer Center, Houston, TX 77030, USA; The Jackson Laboratory for Genomic Medicine, Farmington, CT 06032, USA; The Jackson Laboratory, Bar Harbor, ME 04609, USA; Seven Bridges, Charlestown, MA 02129, USA; Seven Bridges, Charlestown, MA 02129, USA; The Jackson Laboratory for Genomic Medicine, Farmington, CT 06032, USA

## Abstract

We created the PDX Network (PDXNet) portal (https://portal.pdxnetwork.org/) to centralize access to the National Cancer Institute-funded PDXNet consortium resources, to facilitate collaboration among researchers and to make these data easily available for research. The portal includes sections for resources, analysis results, metrics for PDXNet activities, data processing protocols and training materials for processing PDX data. Currently, the portal contains PDXNet model information and data resources from 334 new models across 33 cancer types. Tissue samples of these models were deposited in the NCI’s Patient-Derived Model Repository (PDMR) for public access. These models have 2134 associated sequencing files from 873 samples across 308 patients, which are hosted on the Cancer Genomics Cloud powered by Seven Bridges and the NCI Cancer Data Service for long-term storage and access with dbGaP permissions. The portal includes results from freely available, robust, validated and standardized analysis workflows on PDXNet sequencing files and PDMR data (3857 samples from 629 patients across 85 disease types). The PDXNet portal is continuously updated with new data and is of significant utility to the cancer research community as it provides a centralized location for PDXNet resources, which support multi-agent treatment studies, determination of sensitivity and resistance mechanisms, and preclinical trials.

## Graphical Abstract

**Graphical Abstract:**
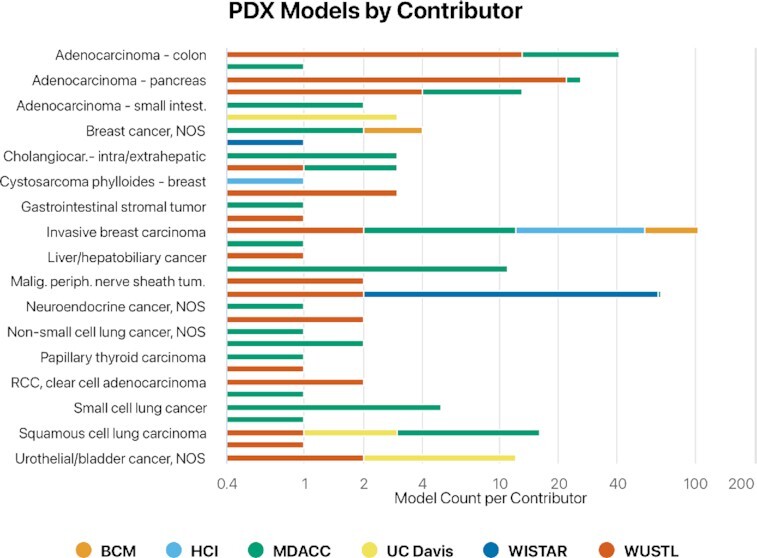
The PDXNet portal provides a way for researchers to learn about the PDX models, sequencing data and PDX Minimum Information metadata tools generated by the network for public use.

## INTRODUCTION

Patient-derived Xenograft (PDX) models are cancer models that support personalized medicine research and preclinical and co-clinical trials (1–5). Specific PDX research areas include the study of sensitivity and resistance mechanisms, evaluation of new treatment options, and the study of tumor heterogeneity. The PDX research community is rapidly growing, with PDX-generated data being the preferred support for proposing human clinical trials (6). In 2017, the National Cancer Institute (NCI) funded the PDX Development and Trial Centers (PDTC) research network (PDXNet, https://www.pdxnetwork.org/) consortium to accelerate PDX research by developing new PDX models across cancer types, identifying new multi-agent treatments to bring forward into clinical trials, generating complementary RNA-Seq and whole-exome sequencing data, and increasing the ethnic diversity of publicly available PDX models.

PDXNet was also charged with developing collaborative research projects involving the six different PDTCs to advance PDX science. Each of the PDTCs came into PDXNet with its own home-grown data standards, data analytic pipelines and workflows. To facilitate collaboration, the disparate processes and databases required harmonization at many different steps, so that data from centers could be combined and analyzed efficiently. The harmonization goal was achieved through the creation of the PDXNet portal and the analytical tools within it. The PDXNet portal resources created by this effort enabled the successful completion of several collaborative research projects (7–10) and are supporting many others.

In addition to facilitating PDXNet research, a further benefit of the PDXNet portal is to make the PDXNet-generated data and workflows of the PDXNet portal available as a public resource. These data will support cancer research by increasing the quantity and diversity of PDX data available and decreasing the effort required to analyze PDX sequencing data. We present the PDXNet portal as a utility for PDXNet data for the larger scientific community.

The PDXNet portal is already supporting the goals of data harmonization, discovery, generation and analysis. To demonstrate the utility of the portal resource, we highlight an in progress PDXNet pilot project. Initial review of model metadata identified the need for standardized ancestry information across the PDTCs. For this project, the analyst accessed the metadata (downloadable from the PDXNet portal and described below) to identify related sequencing data from PDX model IDs. For each available model, the analyst then generated standardized ancestry estimates with a published ancestry inference workflow (see result section for details). These ancestry estimates were then added to the portal metadata in a searchable interface. Moreover, we created a portable and reproducible version of the publish ancestry inference workflow, allowing other researchers to use and independently validate once linked to the PDXNet Portal.

There are several existing public PDX resources that complement the PDXNet portal. Launched in 2012, the NCI Patient-Derived Model Patient Repository (PDMR; 11) collects and develops PDX models and associated standardized sequencing data (RNA-Seq and whole-exome), with the goal of supporting academic and industry research. The PDMR maintains a publicly available database of models and a File Transfer Protocol (FTP) site for accessing sequencing data (https://pdmr.cancer.gov/database/default.htm). Another resource is PDXFinder (https://www.pdxfinder.org, 12). PDXFinder is an online resource that aims to harmonize internationally generated PDX models and their associated metadata. PDXFinder is a collaboration between the European Molecular Biology Laboratory’s European Bioinformatics Institute (EMBL-EBI) and the Jackson Laboratory. A key component of data harmonization in PDXFinder is the PDX minimal information standard (PDX-MI; 13), which allows for standardized PDX information exchange. PDXFinder employs PDX-MI to support complex model searches that enable users to identify model descriptions and subsequently link to model information and a request form. EuroPDX is a consortium of eighteen non-profit cancer institutes that collaborate and coordinate PDX model development and access to improve cancer patients’ standard of care. Next, the EuroPDX Data Portal (https://dataportal.europdx.eu/) is a resource that provides information about PDX models generated by EuroPDX researchers and clinicians. Lastly, the Baylor College of Medicine PDX portal (https://pdxportal.research.bcm.edu/) provides access to breast cancer, leukemia, pediatric liver cancer, pancreatic cancer and sarcoma model collections.

The primary aim of the PDXNet portal is to support the Cancer Moonshot (14) model and data sharing goals (15). The PDXNet portal facilitates the distribution of resources, complementary data analyses and developed tools. The resources generated include data collections and standardized bioinformatics workflows. Complementary data analyses such as data quality control analyses that support data-use are also available from the portal. Tools developed to support the use of the data (e.g. workflow cost estimation) are also accessible. Integration with the NCI Cloud Resource, the Cancer Genomics Cloud powered by Seven Bridges (CGC; 16), allows approved researchers to directly analyze PDXNet data or use developed workflows on private data.

This manuscript details the PDXNet portal features that provide a gateway for identifying and accessing resources generated by the PDXNet community.

## MATERIALS AND METHODS

### Portal features

The PDXNet portal is a publicly accessible website (https://portal.pdxnetwork.org/) with the primary function of providing access to the PDXNet models and information on how to obtain sequencing data. We extended the portal’s primary mission beyond PDXnet to incorporate additional resources, including supporting access to the PDMR sequencing file data set, a PDXNet hematoxylin and eosin stain image data set, and tumor volume data. Below, we describe the features and sections of the Portal in detail.

### PDXNet portal landing page

**Figure 1.**
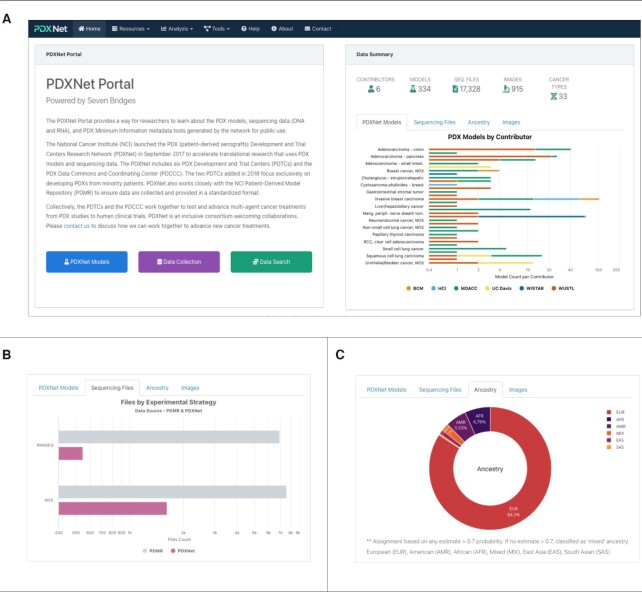
PDXNet Portal Landing Page Views from the PDXNet Portal Landing Page. (**A**) The initial PDXNet Portal Landing Page. (**B**) Experimental strategies (whole-exome and RNA-Seq) for the PDXNet (magenta) and PDMR (gray) sequencing datasets. (**C**) Computed ancestry in a pie chart. Ancestry is classified in the following categories: European (EUR-Red), African (AFR-Blue), American (AMR-Purple), Mixed (MIX-Orange), East Asian (EAS-Light Purple), South Asian (SAS-Yellow).

The PDXNet portal landing page includes an overview and summary panel of contents (Figure 1). The portal overview identifies the primary PDXNet funding sources and participants. In summary, the NCI Cancer Therapy Evaluation Program (CTEP) funds four PDTCs and the PDX Data Commons and Coordinating Center (PDCCC), whereas the NCI Center to Reduce Cancer Health Disparities funds two PDTCs (see Table 1 for additional details). The portal directs questions and requests for additional information to the PDXNet website at https://pdxnetwork.org.

**Table 1.** PDXNet Development and Trial Centers (PDTC) and the PDX Data Commons and Coordinating Center (PDCCC)

PDXNet	
**PDX Development and Trials Centers (PDTC)**	
HCI-BCM*	Huntsman Cancer Institute and Baylor College of Medicine
MDACC	MD Anderson Cancer Center
WUSTL*	Washington University at St. Louis
WISTAR*	Wistar Institute and MD Anderson Cancer Center
BCM&	Baylor College of Medicine
UCDAVIS^&^	The University of California at Davis
**PDX Data Commons and Coordinating Center**	
JAX-SB^*&^	Jackson Laboratory and Seven Bridges

*NCI Cancer Therapy Evaluation Program Funding

^&^NCI Center to Reduce Cancer Health Disparities Funding

The data summary panel on the right side of the screen allows the reader to review model and data summaries and the portal update timeline. The data summary panel lists the number of PDTCs contributing data, the number of files uploaded by the PDTCs and available from the PDMR, the total number of models, and the number of cancer types represented in those models. Dropdown menus on the top bar of the landing page include ‘Resources’, ‘Analysis’, ‘Tools’, ‘Help’, ‘About’ and ‘Contact’, which are further summarized below. Buttons at the bottom left provided quick access to PDXNet model information, data resources, and data search. Tabs associated with the right-hand data summaries allows the reader to review summary figures for PDX Models by cancer type and contributing PDTC, sequencing files by experimental strategy, ancestry and the Portal Update Timeline.

### Resources tab

The PDXNet Portal Resources tab includes the following links to separate pages: PDXnet models, Data Collection, Interactive Exploration, and Workflows, and subpages are described in subsequent sections.

#### PDXNet models

The PDXNet portal models tab summarizes verified model submissions to the PDMR made by each PDTC (Supplementary Figure S1). The PDXNet models are a primary consortium deliverable. Each model submitted by a PDTC to the PDMR includes a completed model submission form that details the general PDX information, model-specific information, and tissue implantation details. Metadata are consistent with the PDX-MI and the PDMR data format. The metadata includes model id information to facilitate search and cross referencing to related PDMR models.

To date, PDXNet researchers have submitted 334 models to the PDMR across 33 cancer types. The most prevalent model cancer types include invasive breast carcinoma (30.8%, 103), melanoma (20.1%, 67), adenocarcinoma—colon (12.3%, 41), and adenocarcinoma—pancreas (7.8%, 26). See Table 2 for additional details.

**Table 2.** PDX models generated by PDX Development and Trials Centers

	HCI-BCM	MDACC	WUSTL	Wistar	UC Davis	BCM	Totals
Breast	78	12	2	0	0	16	108
Head and Neck	0	0	2	0	0	0	2
Digestive/Gastrointestinal	0	50	44	0	3	0	97
Endocrine and Neuroendocrine	0	2	0	0	0	0	2
Musculoskeletal	0	1	4	0	0	0	5
Respiratory/Thoracic	0	30	1	0	2	0	33
Skin	0	3	2	63	0	0	68
Genitourinary	0	0	5	0	10	0	15
Gynecologic	0	3	0	0	0	0	3
Unknown Primary	0	0	0	1	0	0	1
**Total**	**78**	**101**	**60**	**64**	**15**	**16**	**334**

*HCI-BCM*: Huntsman Cancer Institute and Baylor College of Medicine, MDACC: MD Anderson Cancer Center, WUSTL: Washington University at St. Louis, Wistar: The Wistar Institute, UC Davis: University of California Davis, BCM: Baylor College of Medicine

#### Data search

The PDXNet Portal data search page allows users to query across data types, and harmonizes metadata across model information, sequencing files and image files. Users query from data source, samples and disease types. Query results are shown in Venn diagrams and users can download data tables organized by sample, model or patient at the bottom of the page. The data search feature specific currently queries across with corresponding PDMR image, RNA-seq and WES data.

#### Data collection

The PDXNet Portal Data Collection tab summarizes sequencing data from the PDMR and PDTCs, and image files.

##### Data collection, PDMR

This sub-tab summarizes sequencing data transferred from the PDMR FTP server to the CGC as of December 2021 (Supplementary Figure S2). The PDMR generates whole-exome and transcriptome sequencing data from models submitted according to tissue collection best practices and model quality control practices (11). Molecular characterizations include whole-exome sequencing and gene expression profiling. We processed the PDMR sequencing data with standardized workflows as for the PDTC data.

##### Data collection, PDTC

**Figure 2.**
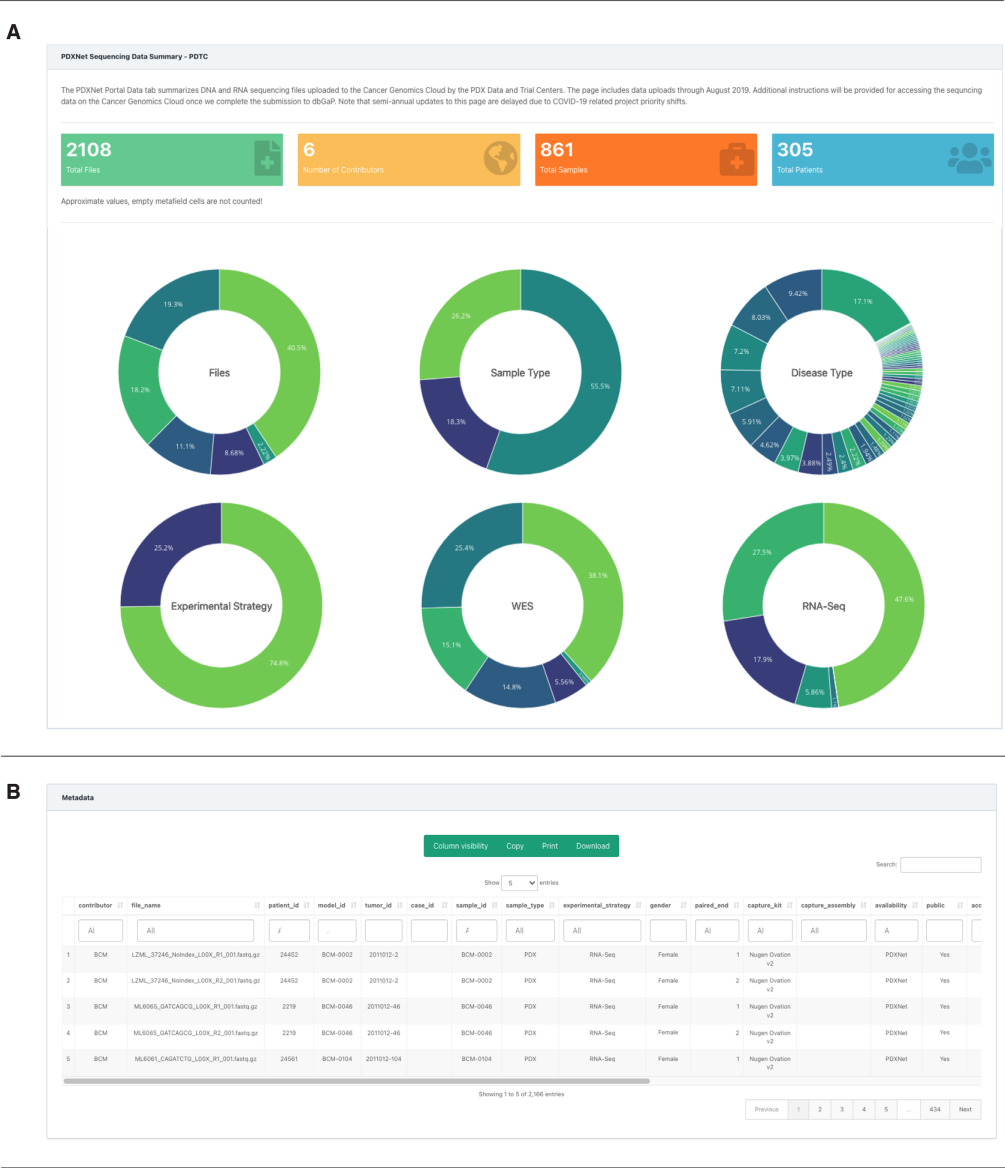
PDXNet sequencing data page on the PDXNet Portal Components of the PDXNet sequencing data page. (**A**) Panel shows summary statistics including number of sequencing files (green), contributors (yellow), total samples (orange) and total patients (blue). Also, shown are donut plots for contributors, sample types, disease type, experimental strategy, WES contributors and RNA-Seq contributors. (**B**) Panel shows metadata for the PDXNet sequencing data in a spreadsheet format. The interface supports searching and sorting metadata. Users can copy, print, and download metadata into accessible formats.

This sub-tab summarizes sequencing data submitted by the PDTCs for intra-consortium sharing and for public sharing (Figure 2). The PDXNet Data Collection, PDTC tab presents the core PDXNet sequencing data set. We processed submitted sequencing data with standardized workflows (e.g. whole exome capture) according to a written standard operating procedure provided on the CGC. See the workflow section for description of the workflows used for standardized processing.

PDXNet researchers contributed 2134 total sequencing files that include both whole-exome (74.5%, 11 590) and RNA-Seq (25.5%, 544) data. Six institutional contributors submitted 873 samples from 308 patients. The sequencing sample types include PDX (56.0%, 1194), tumor (25.7%, 548) and normal (18.4%, 392). The most prevalent diseases represented among the samples include breast (27.5%, 586), skin (18.8%, 402), lung (17.1%, 364) and colon (8.4%, 180). Metadata provided by centers did not include disease information for 0.9% (20) samples (see Table 3 for additional information).

**Table 3.** Sequencing data files generated by PDX Development and Trial Centers

	Overall	BCM-HCI	MDACC	UC Davis	WISTAR	WUSTL
	*N* = 2134	*N* = 408	*N* = 418	*N* = 48	*N* = 382	*N* = 878
**Experimental strategy *n* (%)**						
RNA-Seq	544 (25%)	102 (25%)	6 (1%)	32 (67%)	144(38%)	260(30%)
WES	1590 (75%)	306 (75%)	412(99%)	16 (33%)	238(62%)	618(70%)
**Disease type, *n* (%)**						
Bladder	114(5.0%)	2(<0.5%)	4(1.0%)	48(100%)	4(1%)	56(6.4%)
Blood	84(4.0%)	0(0%)	84(20.0%)	0(0%)	0(0%)	0(0%)
Bone	8(0.4%)	0(0%)	0(0%)	0(0%)	0(0%)	8(0.9%)
Breast	586(27%)	382(93%)	0(0%)	0(0%)	0(0%)	204(23%)
Colon	180(8.4%)	4(1.0%)	8(2.0%)	0(0%)	0(0%)	160(18%)
Gastrointestinal	34(1.6%)	0(0%)	0(0%)	0(0%)	0(0%)	34(3.9%)
Head and Neck	24(1.1%)	0(0%)	0(0%)	0(0%)	0(0%)	24(2.7%)
Kidney	58(2.7%)	0(0%)	0(0%)	0(0%)	0(0%)	58(6.6%)
Lung	364(17%)	0(0%)	322(77%)	0(0%)	0(0%)	42(4.8%)
Ovarian	8(0.4%)	0(0%)	0(0%)	0(0%)	0(0%)	8(0.9%)
Pancreas	174(8.1%)	0(0%)	0(0%)	0(0%)	0(0%)	174(20%)
Rectum	22(1.0%)	0(0%)	0(0%)	0(0%)	0(0%)	22(2.5%)
Skin	402(19.0%)	0(0%)	0(0%)	0(0%)	370(97%)	32(3.7%)
Soft Tissue Neoplasm	56(2.6%)	0(0%)	0(0%)	0(0%)	0(0%)	56(6.4%)
Unknown	20(0.9%)	20(5.0%)	0(0%)	0(0%)	0(0%)	0(1.7%)
**Sample type, *n* (%)**						
Normal	392(18%)	32(8%)	142(34%)	0(0%)	0(0%)	218(25%)
PDX	1,194(56%)	328(80%)	146(35%)	0(0%)	202(53%)	515(59%)
Tumor	548(26%)	48(12%)	130(31%)	48(100)	180(47%)	142(16%)

*HCI-BCM*: Huntsman Cancer Institute and Baylor College of Medicine, *MDACC*: MD Anderson Cancer Center, *WUSTL*: Washington University at St. Louis, *Wistar*: The Wistar Institute, *UC Davis*: University of California Davis, *BCM*: Baylor College of Medicine

The PDMR sequencing dataset on the CGC includes 14 506 paired-end sequencing files that include both whole-exome (52.2%, 7576) and RNA-Seq (47.8%, 6926) data. The data set includes 3857 samples from 629 patients covering 85 disease types. The sequencing sample types include PDX (81.4%, 11 808), primary tumor (5.6%, 812), pdc: mixed tumor culture (4.7%, 684) and normal germline (4.4%, 644). The most prevalent diseases represented among the samples include colon (19.2%, 2,780), pancreas (9.2%, 1,332), melanoma and (6.5%, 938). Due to the size and cost associated with data transfers, synchronization between the PDMR sequencing database and the CGC dataset is done periodically. The PDMR data webpage has the most updated list of available PDMR sequencing data processed with standardized PDXNet workflows.

##### Data collection, Image

This sub tab summarizes hematoxylin-eosin stain (H&E) image data provided by the PDMR (Supplementary Figure S3). The PDMR image data on the CGC includes 194 images scanned from PDX (96.4%, 187) and primary tumors (3.6%, 7). The images correspond to 194 samples taken from 27 patients across 4 disease types. The PDX passages ranged from P0 to P6 with the top four passages corresponding to P1(40.6%, 73), P0 (25.0%, 45), P2 (22.8%, 41) and P3 (7.2%, 13). The PDXNet Portal currently supports 43 metadata fields that data submitters can populate upon submission (See Supplementary Table S1 for the complete list).

#### Interactive exploration

The PDXNet Portal data explorer allows users to interactively create summary tables from PDMR and PDTC sequencing file metadata (Supplementary Figure S4). The interactive table supports 10 table and chart types including simple tables, bar charts, line charts, and heat maps (see Supplementary Table S2 for full list). Interactive tables also support 22 data summary options including count, sum, average and variance (see Supplementary Table S3 for the full list). The user drags and drops from 20 metadata fields to the table type area to construct the table. Metadata field examples include contributor, sample type, experimental strategy and passage (see Supplementary Table S4 for complete list).

#### Workflows

The PDXNet Portal Workflows page summarizes analysis workflows developed by the PDXNet community (Supplementary Figure S5). The CGC based analysis workflows are a significant resource developed by the PDXNet community, allowing for reproducible and standardized analysis of PDX data (8). We selected workflows for standard consortium-wide data processing and public release from those submitted by each PDTC after benchmarking with simulated and experimentally derived PDX data (8). Since the initial public release, we have restructured the workflows to efficiently process normal (tissue), tumor-only and tumor-normal data. These workflows are implemented on the CGC using the Common Workflow Language (CWL; 17) with Docker containerized tools, which allows for easy sharing and analysis reproducibility. The PDXNet consortium developed a set of 15 workflows validated for processing of both whole-exome and RNA-Seq data (See Supplementary Table S5). We are sharing these validated and tested workflows with the broader community via the CGC Public Apps Gallery (https://cgc.sbgenomics.com/public/apps#q?search=pdx). The use of CWL allows these workflows to be portable to any CWL-compliant execution environment. The workflows collectively facilitate the analysis of whole-exome or RNA-Seq data via mouse read disambiguation, read alignment, variant calling or transcript quantification, and sample and cohort level quality control. For whole-exome data, we also compute copy number variation (CNV), tumor mutational burden (TMB), microsatellite instability (MSI) and homologous recombination deficiency (HRD) during standardized processing. A full explanation of inputs, outputs and data processing steps for each workflow is provided on the CGC in the respective description section.

### Analysis tab

The PDXNet Portal Analysis tab includes the following menu options: Ancestry Analysis, HRD-MSI-TMB Analysis, Tumor Volume Analysis, QC Analysis and Variant Search. These sections include metrics derived from primary data sources and are described in the results section below in more detail. These results were generated from standard processing analysis workflows or through PDXNet research activities (8), and we provide these analyses to support independent research by the broader research community.

### Tools tab

The PDXNet Portal Tools tab includes the following choices: Workflow Cost Prediction, Metadata -Create, Metadata -Validate. These tools, described in the following sections, support present and future PDXNet and other independent general research activities.

#### Workflow cost prediction

The Workflow cost prediction tool allows users to estimate the cost of processing their samples on the CGC with the PDXNet workflows. This tool uses prediction models (gradient boosting trees; 18) generated from 7000 workflow runs. The user can select either whole-exome or RNA-Seq workflows and provide the number and optionally size of files to process. The calculator computes the storage and computation cost for processing the user defined dataset. The estimated costs assume the workflows were run on the CGC using spot instances. We expect the tool to allow users to estimate data storage and computational cost for their own analyses allowing for estimating grant budgets and budgeting lab expenses.

#### Create

The generate metadata tab allows users to interactively generate a PDX minimum information metadata sheet (PDX-MI). As described above, the PDXFinder working group developed the PDX-MI as a standard for exchanging PDX information among institutions. The generate metadata tab allows the user to create a PDX-MI spreadsheet by stepping through data entry dialog boxes. The interface supports entry of patient information, treatment information, tumor information, model and sequencing metadata. The user downloads the spreadsheet upon data entry completion, and no information is stored permanently on the PDXNet Portal site.

#### Metadata, validate

The validate metadata tab allows users to upload and validate a PDX-MI metadata spreadsheet. The user can review uploaded contents at the bottom of the page. The validation verifies that required metadata fields are present and that entries are valid. The validation feature generates a summary of required fields that includes percent completed and most common data entry per field. The validation feature reduces the amount of time necessary to review and check submitted PDX-MI spreadsheets.

### Portal design and organization

The PDXNet Portal is designed to support coordination with other resources, including the PDMR, the CGC and the NCI Cancer Data Service (CDS; https://datacommons.cancer.gov/). Currently, the PDXNet portal references PDMR model information, genomic, transcriptomic and tumor volume response data used in PDXNet research activities. The CGC serves as a PDXNet data staging area supporting data harmonization, standardized data processing and research activities. The PDXNet Portal supports submission of sequencing data to the CDS to provide long-term research access to PDXNet data resources. The CDS is part of the NCI Cancer Research Data Commons which aims to store data resources generated by NCI-funded research. The CDS is available from across the NCI data infrastructure through a dbGaP access mechanism. The PDXNet portal augments the dbGaP submission process, through an administrative feature for generating data reports and through a dbGaP submission tool written to support CDS submissions. The PDXNet Portal aims to use data standards when they exist, supporting both the PDX-MI standard (13) and the PDMR data structures (11). These existing data structures allow for collaboration and information sharing with existing PDX resources.

### Implementation

The backend of the PDXNet Portal is an R-Shiny app hosted on a cloud-based server. The portal uses the PDMR and PDX-MI metadata standards. The PDMR and PDX-MI standards allow us to harmonize data across sources, quickly import data from the PDMR and other data sources, and exchange information with other PDX related portals. We also collect additional metadata required to facilitate computation on the CGC, including omics-related information. We use the Cancer Therapy Evaluation Program (CTEP) disease classification to standardize disease entries although we initially accepted institutionally defined disease classification. In the cases where a standard does not exist; we collect sufficient metadata required to display and process the data source. For example, we take a minimalistic approach to managing tumor volume and H&E image data. Several PDXNet teams are working toward the development of best practices for these data types. Until these best practices are published, we will evolve these operational standards to support harmonization and analysis.

The PDXNet Portal team updates information on the portal semi-automatically using the same data model as the PDMR, allowing PDXNet to sync with PDMR model information. The PDMR provides regular updates to PDXNet on PDTC model submissions to update the PDX Model’s page. We receive sequencing data upload updates from the PDTCs and the PDMR, and we have developed scripts for extracting PDXNet standardized processing results, allowing for quality control information and computational metrics to be tabulated for semi-automated PDXNet Portal updates. The PDXNet Portal source code will not be made publicly available for security reasons. Future PDXNet Portal versions will support controlled access sign-in to provide links to controlled files.

## RESULTS

All analysis subsections described below can be found as selections under the ‘Analysis’ tab in the menu bar of the portal page.

### Ancestry analysis

The PDXNet Portal Ancestry Analysis page summarizes genetic ancestry analysis for datasets on the portal (Supplementary Figure S6). We compute ancestry with SNPweight (19) using a reference dataset generated from the 1000 Genomes Project Phase III (20). We classify each sample into one of five categories, which correspond roughly to the concept of ‘continental ancestry’ (21). These categories include European (EUR), African (AFR), American (AMR), East Asia (EAS) and South Asian (SAS). Samples that could not be confidently assigned to one of these categories are labeled Mixed (MIX). On the left side of the page, ancestry data filters allow the user to select the data contributors, ancestry and disease type. Applying the selected filter to the data regenerates the two summary figures. The first summary figure is a bar chart that shows the ancestry distribution for the selected disease types. The second summary figure shows a pie chart with each slice corresponding to the ancestry types chosen. Supplementary Table S6 shows the summary of ancestry estimation from PDX Models submitted to the PDMR.

### HRD-TMB-MSI analysis

**Figure 3.**
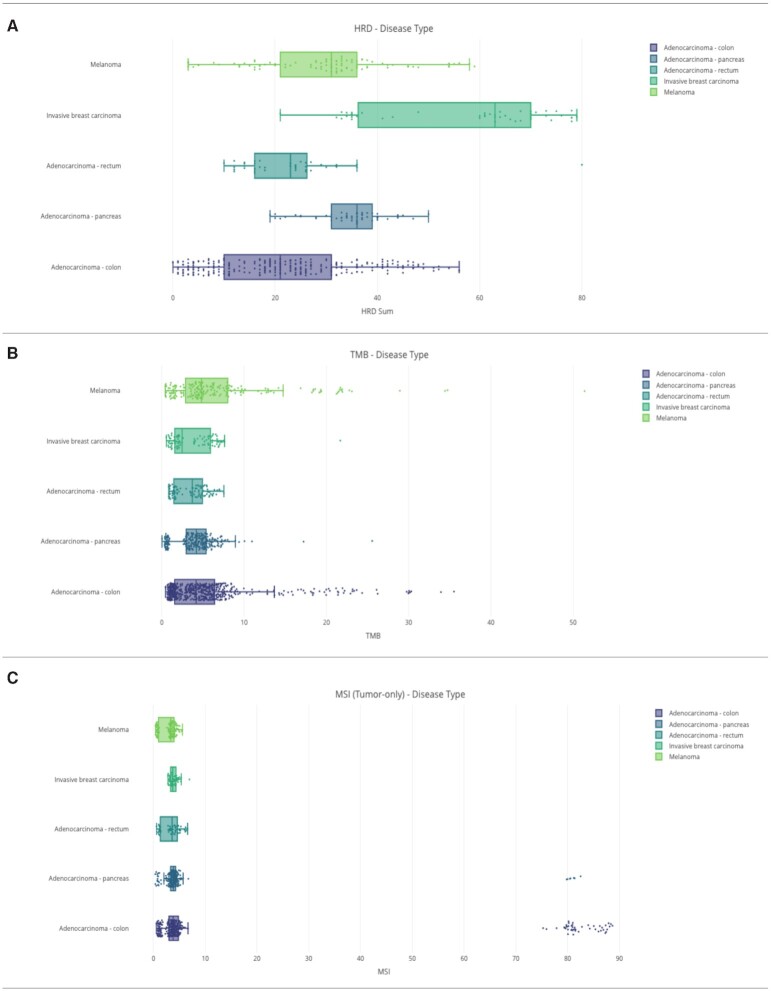
Examples figures generated from the HRD-TMB-MSI page on the PDXNet Portal Plots generated on the PDXNet Portal HRD-TMB-MSI page. (**A**) Plot of Homologous Recombination Deficiency (HRD) computed from sequencing data provided by PDXNet researchers. The plot shows HRD by disease type. (**B**) Plot of Tumor Mutational Burden (TMB) computed from sequencing data provided by PDXNet researchers. The plot shows TMB by disease type. (**C**) Plot of TMB computed from sequencing data provided by PDXNet researchers, by disease type.

The PDXNet Portal HRD-HSI-TMB analysis page allows the user to filter and summarize three computational metrics generated from whole-exome sequencing data by PDXNet standardized processing (Figure 3). The three computed metrics are homologous recombination deficiency (HRD), tumor mutational burden (TMB) and microsatellite instability (MSI). HRD is computed with ScarHRD (22) for matched normal data. TMB is calculated as the number of coding mutations that meet all quality criteria per Mb of the genome. Quality criteria are assessed using coverage, allele frequency, mapping quality and strand bias. Variants included in the calculation are somatic and non-polymorphic, and are defined in SnpEff (23) as ‘high’ or ‘moderate’ functional impact. As only a portion of the genome was sequenced, genome coverage (Mb) is calculated from the input target coverage BED file. MSI is calculated with MANTIS (24) for samples with matched normal data and calculated with MSIsensor2 (25) for tumor-only samples. For each metric, users can set data filters for the visualizations. The data filters, on the left side of the page, allow the user to select data contributor, sample type, experimental strategy and disease type. Applying the selected filter to the data generates a boxplot chart displaying the selected metrics for each disease type chosen (Figure 3).

### Tumor volume analysis

**Figure 4.**
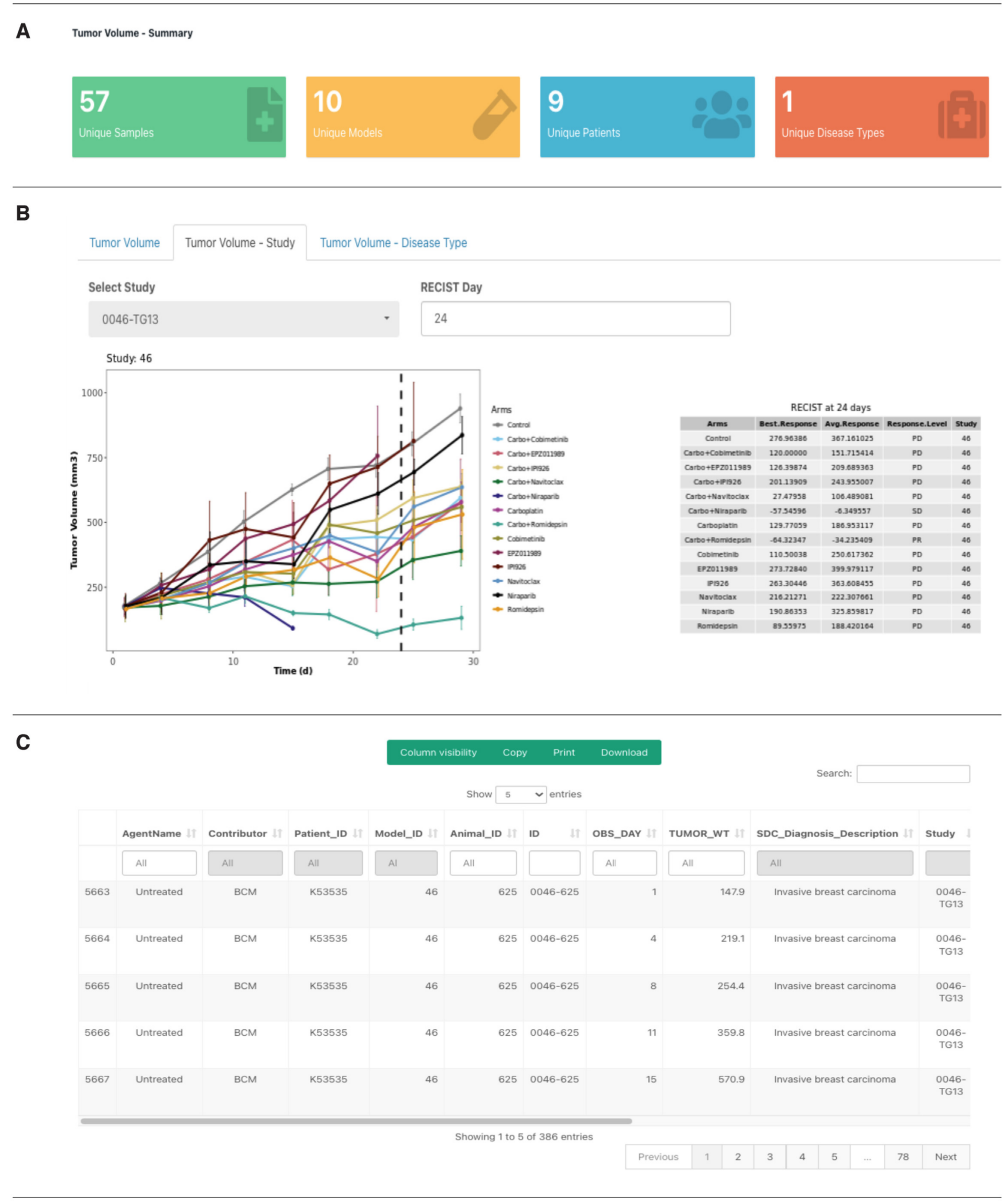
Tumor volume data page on the PDXNet Portal Components of the PDXNet tumor volume page. The figure shows a filtered dataset. (**A**) Panel shows summary statistics including number tumor volume datasets (green), number of models in the selected dataset (yellow), total number of patients (blue), and total number of diseases represented (red). (**B**) Panel shows the tumor volume data organized by study and automatically computed resist category for each treatment arm. (**C**) Panel shows the tumor volume response and metadata in a spreadsheet format. The interface supports searching and sorting metadata. Users can copy, print, and download metadata into accessible formats

The PDXNet Portal Tumor Volume Analysis Page allows the user to visualize raw tumor volume growth data provided by the PDTCs (Figure 4). The filters enable the user to select contributor, treatment and disease type on the page’s left side. Applying the selected filter to the data regenerates the Tumor Volume and the Tumor Disease Types figure tabs. The currently available volume data is from 20 models representing 1 disease type, which were treated with 17 possible agents. The dataset has 10 432 volume measures from 20 treatment studies. The Tumor Volume tab allows the user to choose plot level (Animal and Treatment Arm) and plot pattern (multiple and combined), reorganizing the plots to correspond to select values. Additionally, under the ‘Tumor Volume – Study’ subtab, users can dynamically generate RECIST response classifications (Figure 4). The RECIST classification calculation is based on combination of the best response and average response adapted from Gao *et al.* (26).

### Quality control analysis

The PDXNet portal QC analysis page provides plots and tabular results for selected QC metrics (Supplementary Figure S7). The page displays QC metrics generated during the standardized data processing procedure for each relevant data type. The page provides sub-tabs showing whole-exome and RNA-Seq quality control metrics for a selected dataset PDXNet or PDMR. The whole-exome tabs plot mean target coverage, percent target bases with >20% coverage, and percent duplication by data contributor. The RNA-Seq tabs plot percent usable bases, percent ribosomal bases and percent correct strand reads. The plotted metrics, along with additional QC metrics are available in a table at the bottom of the page.

### Variant search

The PDXNet Portal Variant Search page allows the user to search for variants derived from whole exome data processed using the PDXNet whole exome pipelines on the CGC. The page allows users to query based on either gene symbol or chromosome position. For each query, a summary of variant types, variants by gene and SNP types is shown. The variants returned in the query are available at the bottom of the page. We currently present all called variants for 491 samples as a beta feature release, with the ability for the user to further filter results as needed.

## DISCUSSION

The PDXNet portal is a vital component of the PDXNet consortium. The portal establishes a mechanism for public discovery of consortium-generated resources including models, data and workflows. The portal allows researchers to examine the data, models and metadata using integrated query features. These portal capabilities facilitate cancer data discovery, a core goal of the NCI Cancer Moonshot program. Additionally, the PDXNet portal allows the consortium to manage data analysis projects by clarifying which data are available, their source, their quality and their suitability for research projects and scientific questions. Within the PDXNet consortium, the PDXNet portal functions as a centralized source of information for the status of the available models. The standardized sequence processing, quality control, and computation of common metrics (e.g. MSI, TMB, HRD and genetic ancestry) further enhance data analysis, model use, prioritization of future models and data collection.

Enhancement of collaboration between researchers is a main objective of the PDXNet portal. To accomplish this, we are integrating the PDXNet portal and PDXNet data with existing NCI, NIH, and NCBI infrastructure. All data visible on the PDXNet Portal will also be available through the Cancer Data Service (CDS) through dbGaP (27) access. Accessing PDXNet data on the Cancer Genomics Cloud (16) allows users to perform sophisticated analyses utilizing cloud computing within an integrated bioinformatics ecosystem. By co-locating data and analysis, as well as integrating data management, this infrastructure can decrease the time required for researchers to perform analyses.

For large consortia such as PDXNet, metadata and secondary data types are often just as important as sequencing data for supporting impactful research. Examples of these additional data types include high-resolution images and tumor volume/drug response data. These data extend the types of problems researchers can address. We expect that the portal’s image and tumor volume functionality will expand as these datasets grow. Future iterations of the portal will include interactive exploration across data types allowing users to address complex research problems.

Although PDXs have caveats as a model system, e.g. the fact that only tumors that successfully engraft can be studied, they are widely used in cancer research. To ensure their optimal use, continuing attention to standards for data submission, access, and quality is essential. The PDXNet Portal reflects PDXNet activities to implement such standards not only for sequencing data but also metadata and secondary data types. Another consideration requiring careful implementation is to balance data security versus ease of use. The PDXNet portal will grow with new data and features as the PDXNet consortium continues to generate new models. Consequently, standardized processing and batch effects are of increasing concern for downstream analyses. To ensure that researchers have confidence in the data quality, we will continue to share the informative metrics computed by the standardized PDXNet quality control workflows.

Several key features are the focus for the next iteration of the PDXNet portal. These include tools to expand our search features for commonly found genomic variants (e.g. INDELs and copy number variations) within models, diseases and genes of interest, and interactive exploration of gene expression data. These tools would enable researchers to perform meaningful analyses directly from the portal and more rapidly realize value from PDXNet data. Visualization and analysis of associated data, including imaging and tumor volume/drug response data, will also be a focus. These data types have the potential for high impact, particularly given the innovation in large scale data visualization techniques in many fields.

Further development of links between the PDXNet portal and NCI computational infrastructure will benefit researchers as well. Moving large quantities of data is time-consuming and can be expensive. Enabling researchers to perform their analysis where the data is already present lowers the entry barrier into the computational analysis of PDX model data. To facilitate these links, we envision users will be able to create cohorts for analysis using the PDXNet portal and transferring that selection to the Cancer Genomics Cloud or other computational platform, where they will be able to easily take advantage of well-developed computational infrastructure.

We will extend PDXNet portal capabilities as the size and complexity of PDXNet datasets grow. These enhancements will allow the research community to quickly find and evaluate PDXNet resources to supplement their research studies. We will continue to improve the PDXNet portal value by collaborating with related PDX initiatives, including the PDMR, PDXFinder and EuroPDX. Such collaborations will demonstrate how to effectively conduct studies across institutions, providing examples for the broader research community in how to optimize their PDX studies with respect to the public PDX models and datasets that are becoming increasingly available.

## DATA AVAILABILITY

Each PDXNet portal data tab allows users to download metadata. Data for smaller data types such as tumor volume data and computed metrics (e.g. HRD and TMB) can be downloaded directly from the portal. For larger data types, please request data from the PDXNet Portal’s Contact page. We will coordinate with PDTCs to make data available either directly or through dbGaP as required by the PDXNet data sharing agreement.

## Supplementary Material

zcac014_Supplemental_File

## APPENDIX

PDXNet Consortium Members:

Matthew Bailey ^1^, Emilio Cortes-Sanchez ^1^, Sandra Scherer ^1^, Chieh-Hsiang Yang ^1^, Maihi Fujita ^1^, Zhengtao Chu ^1^, Ling Zhao ^1^, Andrew Butterfield ^1^, Argun Akcakanat ^2^, Gao Boning ^2^, Kurt Evans ^2^, Bingliang Fang ^2^, Don Gibbons ^2^, Vanessa Jensen ^2^, Dara Keener ^2^, Michael Kim ^2^, Scott Kopetz ^2^, Mourad Majidi ^2^, David Menter ^2^, John Minna ^2^, Hyunsil Park ^2^, Fei Yang ^2^, Brenda Timmons ^2^, Jing Wang ^2^, Shannon Westin ^2^, Timothy Yap ^2^, Jianhua Zhang ^2^, Ran Zhang ^2^, Min Jin Ha ^2^, Huiqin Chen ^2^, Yuanxin Xi ^2^, Luc Girard ^2^, Erkan Yucan ^2^, Bryce P Kirby ^2^, Bingbing Dai ^2^, Yi Xu ^2^, Alexey Sorokin ^2^, Kelly Gale ^2^, Jithesh Augustine ^2^, Stephen Scott ^2^, Ismail Meraz ^2^, Dylan Fingerman ^3^, Andrew Kossenkov ^3^, Qin Liu ^3^, Min Xiao ^3^, Jayamanna Wickramasinghe ^3^, Haiyin Lin ^3^, Eric Ramirez-Salazar ^3^, Kate Nathanson ^4^, Mike Tetzlaff ^2^, George Xu ^5^, Vashisht G. Yennu-Nanda ^6^, Rebecca Aft ^7^, Jessica Andrews ^7^, Alicia Asaro ^7^, Song Cao ^7^, Feng Chen ^7^, Sherri Davies ^7^, John DiPersio ^7^, Ryan Fields ^7^, Steven Foltz ^7^, Katherine Fuh ^7^, Kian Lim ^7^, Jason Held ^7^, Jeremy Hoog ^7^, Reyka G. Jayasinghe ^7^, Yize Li ^7^, Jinqin Luo ^7^, Cynthia Ma ^7^, Jay Mashl ^7^, Chia-Kuei Mo ^7^, Fernanda Rodriguez ^7^, Hua Sun ^7^, Nadezhda V. Terekhanova ^7^, Rose Tipton ^7^, Brian VanTine ^7^, Andrea Wang-Gillam ^7^, Mike Wendl ^7^, Yige Wu ^7^, Matt Wyczalkowski ^7^, Lijun Yao ^7^, Daniel Cui Zhou ^7^, Matthew Ellis ^8^, Michael Ittmann ^8^, Susan Hilsenbeck ^8^, Bert O’Malley ^8^, Amanda Kirane ^9^, May Cho ^9^, David Gandara ^9^, Jonathan Reiss ^9^, Tiffany Le ^9^, Ralph De Vere White ^9^, Cliff Tepper ^9^, David Cooke ^9^, Luis Godoy ^9^, Lisa Brown ^9^, Marc Dall’Era ^9^, Christopher Evans ^9^, Rashmi Verma ^9^, Sepideh Gholami ^9^, David J Segal ^9^, John Albeck ^9^, Edward Pugh ^9^, Susan Stewart ^9^, David Rocke ^9^, Hongyong Zhang ^10^, Nicole Coggins ^10^, Ana Estrada ^10^, Ted Toal ^10^, Alexa Morales ^10^, Guadalupe Polanco Echeverry ^10^, Sienna Rocha ^10^, Ai-Hong Ma ^9,11^

- Huntsman Cancer Institute, Salt Lake City, UT, USA
- The University of Texas M.D. Anderson Cancer Center, Houston, TX, USA
- The Wistar Institute, Philadelphia, PA, USA
- Abramson Cancer Center, The University of Pennsylvania, Philadelphia, PA, USA
- Hospital of the University of Pennsylvania, Philadelphia, PA, USA
- Department of Melanoma Medical Oncology, MD Anderson Cancer Center, Houston, TX, USA
- Washington University School of Medicine, St. Louis, MO, USA
- Baylor College of Medicine, Houston, TX, USA
- UC Davis Comprehensive Cancer Center, University of California – Davis, Davis, CA, USA
- UC Davis Comprehensive Genomics Center, University of California – Davis, Davis, CA, USA
- Jamaica Plains VA Medical Center, Veteran Administration, Jamaica Plains, MA, USA
