# Correction to ‘PDXNet portal: patient-derived Xenograft model, data, workflow and tool discovery’

**DOI:** 10.1093/narcan/zcaf019

**Published:** 2025-05-27

This is a correction to: Soner Koc, Michael W Lloyd, Jeffrey W Grover, Nan Xiao, Sara Seepo, Sai Lakshmi Subramanian, Manisha Ray, Christian Frech, John DiGiovanna, Phillip Webster, Steven Neuhauser, Anuj Srivastava, Xing Yi Woo, Brian J Sanderson, Brian White, Paul Lott, Lacey E Dobrolecki, Heidi Dowst, PDXNet Consortium, Yvonne A Evrard, Tiffany A Wallace, Jeffrey A Moscow, James H Doroshow, Nicholas Mitsiades, Salma Kaochar, Chong-xian Pan, Moon S Chen, Luis Carvajal-Carmona, Alana L Welm, Bryan E Welm, Michael T Lewis, Ramaswamy Govindan, Li Ding, Shunqiang Li, Meenhard Herlyn, Michael A Davies, Jack Roth, Funda Meric-Bernstam, Peter N Robinson, Carol J Bult, Brandi Davis-Dusenbery, Dennis A Dean, Jeffrey H Chuang, PDXNet portal: patient-derived Xenograft model, data, workflow and tool discovery, NAR Cancer, Volume 4, Issue 2, June 2022, zcac014, https://doi.org/10.1093/narcan/zcac014

PDXNet Portal has migrated to the following url: https://pdxnet.shinyapps.io/portal/

This correction does not affect the results, discussion and conclusions presented in the article. These details have been corrected only in this correction notice to preserve the published version of record.

