# Supplementary material for: PDXNet portal: patient-derived Xenograft model, data, workflow and tool discovery
Source: NAR Cancer. 2022 Apr 22;4(2):zcac014. doi: 10.1093/narcan/zcac014 (PMC9026194; doi:10.1093/narcan/zcac014)
Supplement: zcac014_Supplemental_File [file zcac014_supplemental_file.docx]

# Supplementary Materials

## PDXNet Member Contribution

### PDXNet Leadership and Data Submission

AW, BDD, BW, CJB, CXP, DAD, FMB, JD, JM, JHC, JR, GW, LCC, LD, MD, MH, MSC, MTW, NM, PNR, SK, SL, TW, YAE

### Data Science, Management and Processing

AS, BJS, BW, CF, SK, MWL, SS, DAD, JG, JHC, SLS, SN, LED, HD, PDXNet Consortium, YAE, XYW

### Portal Development

AS, CF, DAD, JG, JHC, MWL, SK

### Portal Integration Planning

SK, MWL, DAD, JG, MR, SLS, SS, JHC

### Writing Manuscript

SK, MWL, DAD, JG, JHC

# Supplementary Tables

Supplementary Table 1. Metadata associated with Hematoxylin and eosin (H&E) Images on the PDXNet Portal.

| **Hematoxylin and Eosin (H&E) Image Metadata** | |
| --- | --- |
| Age | Percent stromal content |
| Cell annotation available? | Percent tumor content |
| Contributor | Primary cancer site |
| CTEP Code | Proteomics |
| Diagnosis Subtype | Race |
| Disease Type | Regional annotation available? |
| Engraftment site | RNA-Seq/ Exp array |
| Ethnicity | Sample ID |
| Gender | Sample Type |
| Histology | SNP array |
| Image file name | Stain |
| Is information of this model already in CGC? | Staining/scanning method available? |
| Magnification | Thumbnail |
| Metastatic site | Treatment |
| Model ID | Treatment information in patient tumor |
| Mouse strain | Treatment information in PDX tumor |
| Note | Tumor Biomarkers |
| Original Species | Tumor differentiation |
| Other pathology notes | Tumor Grade |
| Passage | Tumor Stage |
| Patient ID | WES/Mutations |
| Percent necrotic content |  |

Supplementary Table 2. Supportive interactive table options on the PDXNet portal

| **Supported Interactive  Tables and Charts** |
| --- |
| Area Chart |
| Col Heatmap |
| Horizontal Bar Chart |
| Horizontal Stacked Bar Chart |
| Line Chart |
| Row Heatmap |
| Scatter Chart |
| Table |
| Table Bar Chart |
| Treemap |

Supplementary Table 3. Data summary options available through interactive tables on the PDXNet Portal

| **Interactive Table Data Summary Options** | | |
| --- | --- | --- |
| Average |  | Median |
| Count |  | Minimum |
| Count as fraction of column |  | Sample variance |
| Count as fraction of row |  | Standard deviation |
| Count as fraction of total |  | Sum |
| Count unique values |  | Sum as fraction of column |
| First |  | Sum as fraction of row |
| Integer sum |  | sum as fraction of total |
| Last |  | sum over sum |
| List unique values |  | 80% lower bound |
| maximum |  | 80% upper bound |

Supplementary Table 4. PDXNet metadata fields available on the Interactive Table Explorer on the PDXNet Portal

| **PDXNet Metadata Fields Available on the Interactive Table Explorer** | | |
| --- | --- | --- |
| Access Level |  | File size |
| Availability |  | Gender |
| Capture Assembly |  | Investigations |
| Capture Kit |  | Is FFPE |
| Case id |  | Model id |
| Comments |  | Paired End |
| Contributor |  | Patient id |
| Created Date and time |  | Platform |
| Data Category |  | Public |
| Data Format |  | Sample ID |
| Data Type |  | Sample type |
| experimental strategy |  | Tumor id |
| File name |  |  |

Supplementary Table 5. Standardized PDXNet bioinformatics workflows linked to the PDXNet Portal

| **Workflow Description** |
| --- |
| **RNA-Seq** |
| Prepare Multi-sample Data |
| PDX RNA Expression Estimation Workflow |
| PDX RNA Expression Estimation Workflow (Single End) |
| RNA Expression Estimation Workflow Patient Tumor |
| RNA Expression Workflow Patient Tumor (Single End) |
| **Whole Exome Sequence** |
| PDX WES CNV (Xenome) Tumor-Normal Workflows |
| PDX WES Tumor-Normal (Xenome) with Variant Calling, CNV Estimation, TMB, MSI, and HRD Scores |
| PDX WES Tumor-Only (Xenome) with Variant Calling, MSI, and TMB Scores |
| WES Tumor-Normal with Variant Calling, CNV Estimation, TMB, MSI, and HRD Scores |
| WES Tumor-Only with Variant Calling, MSI, and TMB Scores |
| WES Tumor-Only from BAM (Variant, MSI, TMB) |
| WES Tumor-Normal from BAM(Variant, CNV, HRD, MSI, TMB) |
| **SNP Array** |
| SNP Array Tumor-Only Workflow for Illumina Infinium Omni 2.5 Exome-8 (Version 1.4) Snp Array |
| **Quality** |
| PDX WES Sample QC |
| PDX Sample QC |

Supplementary Table 6. Summary of computed ancestry for PDXNet Models

|  | **Contributors** | | | | **# of** |
| --- | --- | --- | --- | --- | --- |
|  | **HCI-BCM** | **MDACC** | **PDMR** | **WUSTL** | **Patients** |
| AFR | 10 | 3 | 8 | 3 | 24 |
| Adenocarcinoma - colon |  |  | 4 |  | 4 |
| Adenocarcinoma - pancreas |  |  |  | 2 | 2 |
| Adenocarcinoma - rectum |  |  | 1 |  | 1 |
| Invasive breast carcinoma | 10 |  |  |  | 10 |
| Lung Adenocarcinoma |  | 3 | 2 |  | 5 |
| Melanoma |  |  | 1 | 1 | 2 |
| AMR | 18 | 2 |  |  | 20 |
| Invasive breast carcinoma | 18 |  |  |  | 18 |
| Lung Adenocarcinoma |  | 2 |  |  | 2 |
| EAS |  |  | 1 |  | 1 |
| Adenocarcinoma - colon |  |  | 1 |  | 1 |
| EUR | 8 | 30 | 158 | 24 | 220 |
| Adenocarcinoma - colon |  |  | 72 | 7 | 79 |
| Adenocarcinoma - pancreas |  |  | 24 | 13 | 37 |
| Adenocarcinoma - rectum |  |  | 14 | 1 | 15 |
| Invasive breast carcinoma | 8 |  | 10 | 2 | 20 |
| Lung Adenocarcinoma |  | 30 | 14 | 1 | 45 |
| Melanoma |  |  | 24 |  | 24 |
| MIX | 1 | 2 | 3 |  | 6 |
| Adenocarcinoma - colon |  |  | 1 |  | 1 |
| Adenocarcinoma - pancreas |  |  | 1 |  | 1 |
| Invasive breast carcinoma | 1 |  |  |  | 1 |
| Lung Adenocarcinoma |  | 2 | 1 |  | 3 |
| **# of Patients** | **37** | **37** | **170** | **27** | **271** |
| HCI-BCM: Huntsman Cancer Center, MDACC: MD Anderson Cancer Center, PDMR: Patient-Derived Model Repository, WUSTL: Washington University at St. Louis | | | | | |

# Graphical User Interface Screenshots

## Resources

**
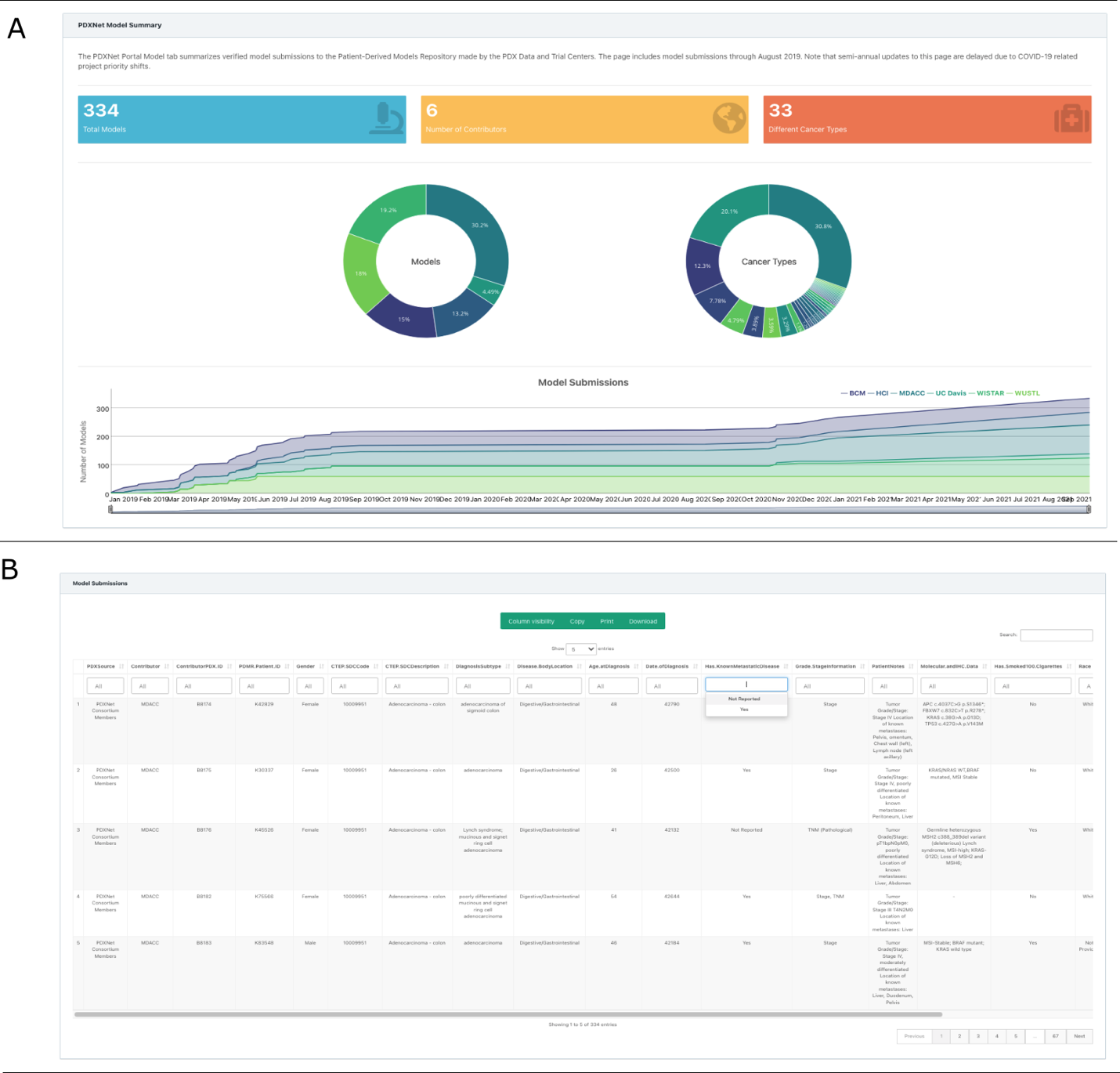
**

Supplement Figure 1. PDX models generated by PDXNet researchers shown on the PDXNet Portal

Figure shows components of the PDXNet Model sequencing data page in separate panels **(A)** Panel shows summary statistics including number of total models (blue), number of contributors (yellow), and number of cancer types (orange). Also, shown are donut plots for contributors and cancer types. Below the donut plots is a chart showing the number of models generated since January 2019. **(B)** Panel shows metadata for the PDXNet PDX models in a spreadsheet format. The interface supports searching and sorting metadata. Users can copy, print, and download metadata into accessible formats.


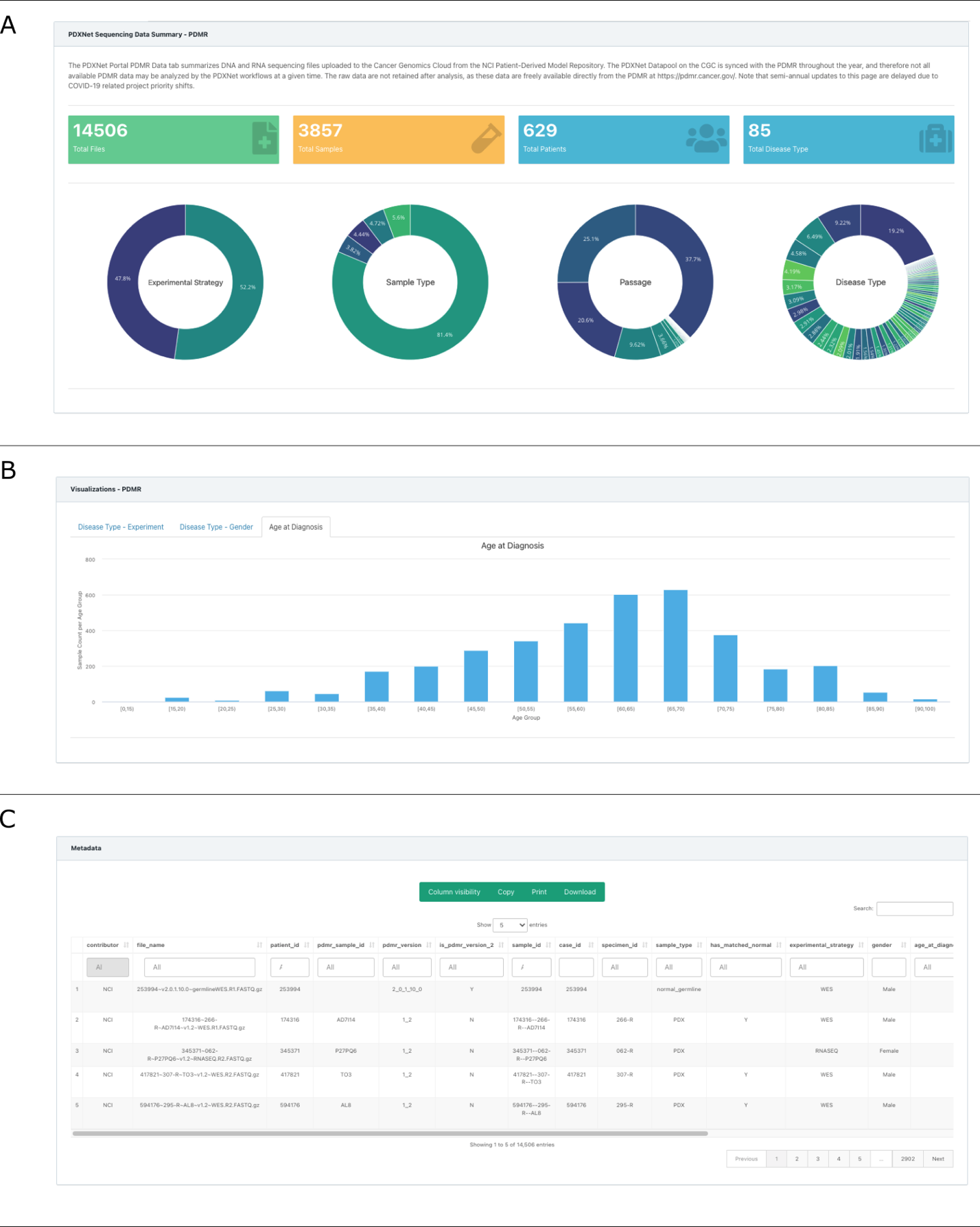


Supplement Figure 2. Patient Derived Model Repository (PDMR) sequencing data listed on the PDXNet portal

Figure shows components of the PDMR sequencing data page in separate panels **(A)** Panel shows summary statistics including number of sequencing files (green), contributors (yellow), total samples (orange), and total patients (blue). Also, shown are donut plots for contributors, sample types, disease type, experimental strategy, WES contributors, and RNA-Seq contributors. **(B)** Panel shows age from PDMR patients on a bar chart. **(C)** Panel shows metadata for the PDMR sequencing data in a spreadsheet format. The interface supports searching and sorting metadata. Users can copy, print, and download metadata into accessible formats.


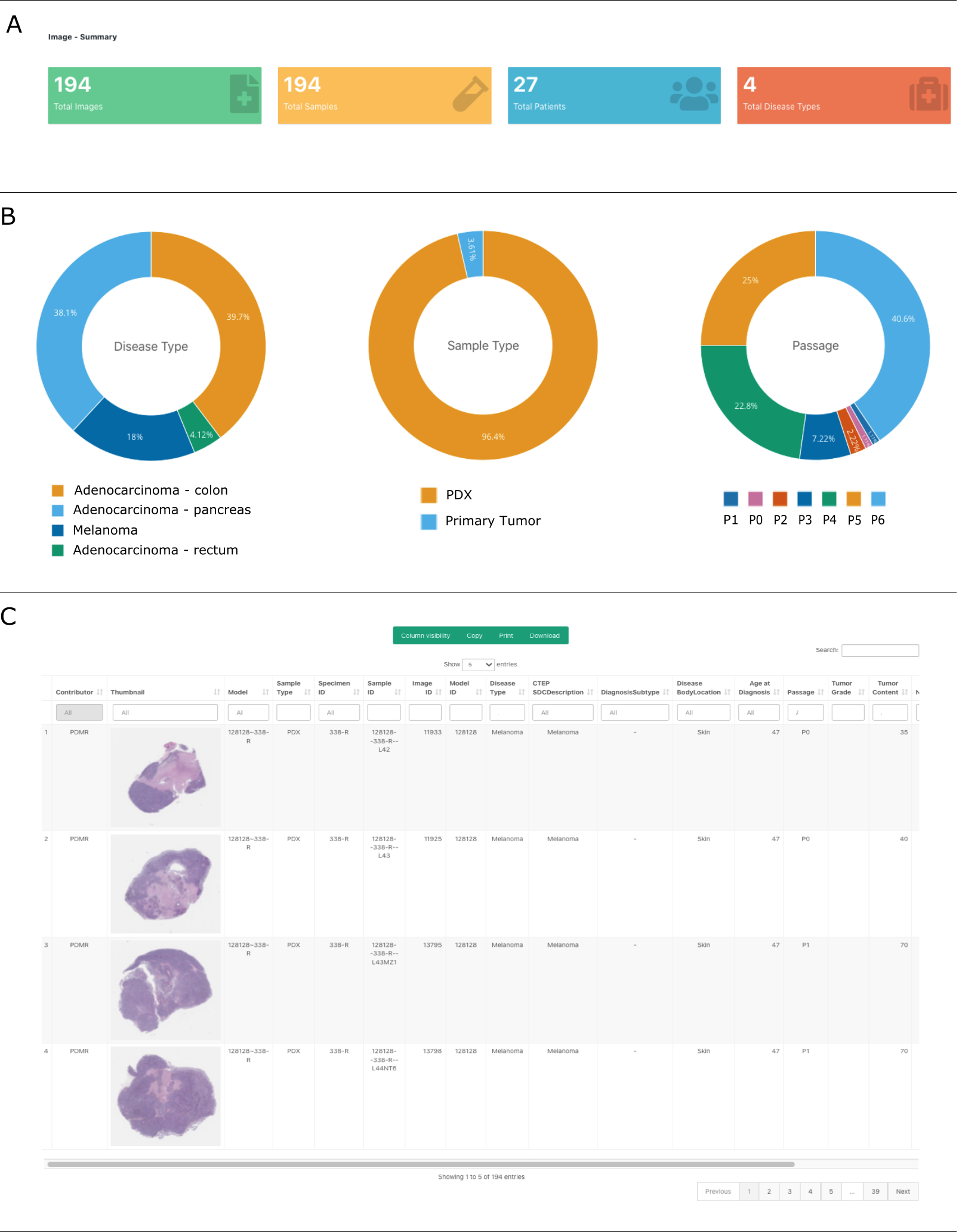


Supplement Figure 3. Patient-Derived Model Repository (PDMR) image page on the PDXNet Portal

Figure shows components of the PDMR image data page in separate panels (A) Panel shows summary statistics including number of images (green), contributors (yellow), total patients (blue), and total disease types (red). (B) Panel B shows donut plots for disease type, sample type, and passage. (C) Panel shows metadata for the PDMR image data in a spreadsheet format. The interface supports searching and sorting metadata. Users can copy, print, and download metadata into accessible formats.


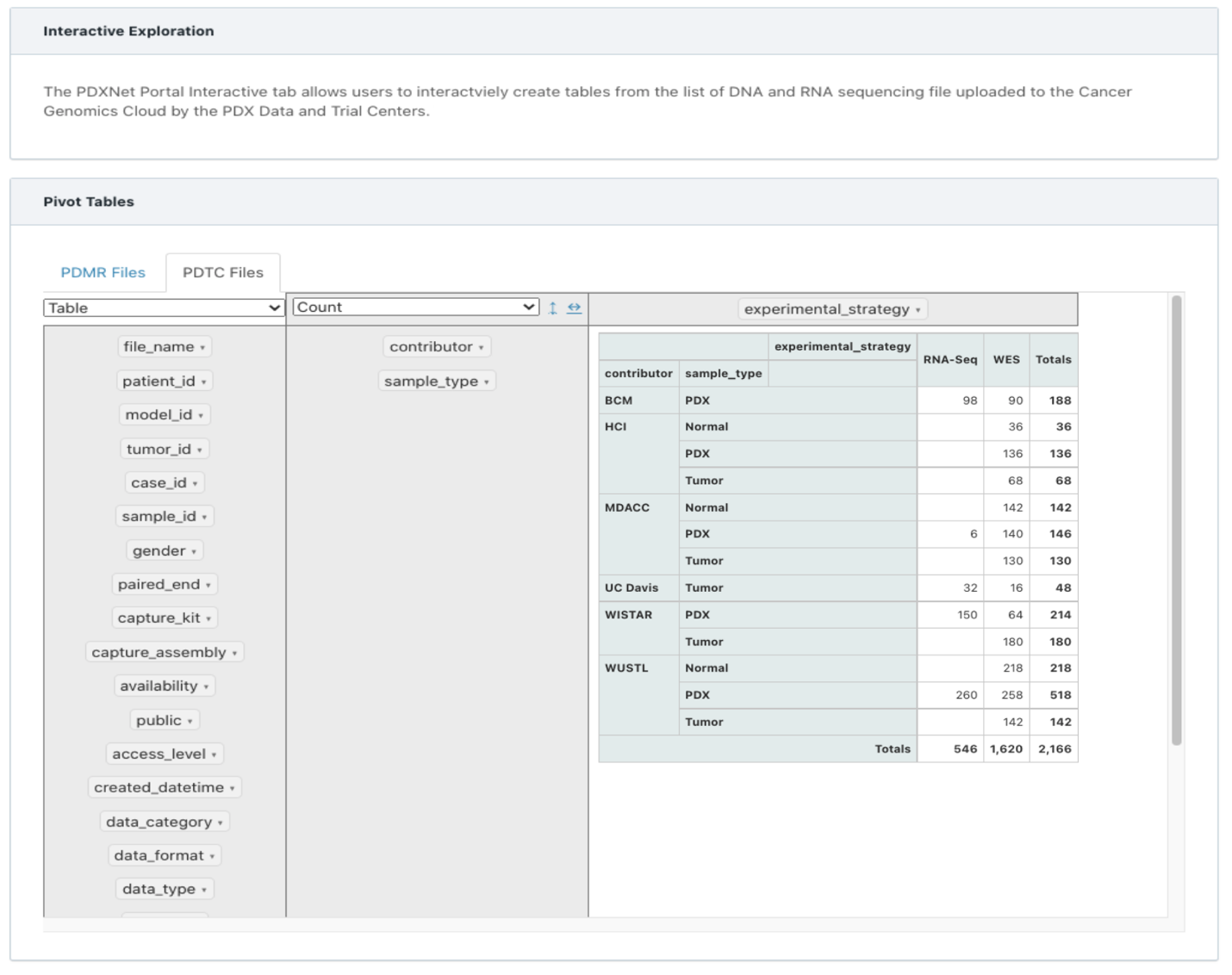


Supplement Figure 4. Interactive data explorer page on the PDXNet Portal

Figure shows the interactive exploration page on the PDXNet portal. The user can interactively create a pivot table with either the metadata from the PDXNet sequencing data or the PDMR sequencing data. Constructing the table involves dragging and dropping table fields on the left side to the table area (green) on the right side of the screen.


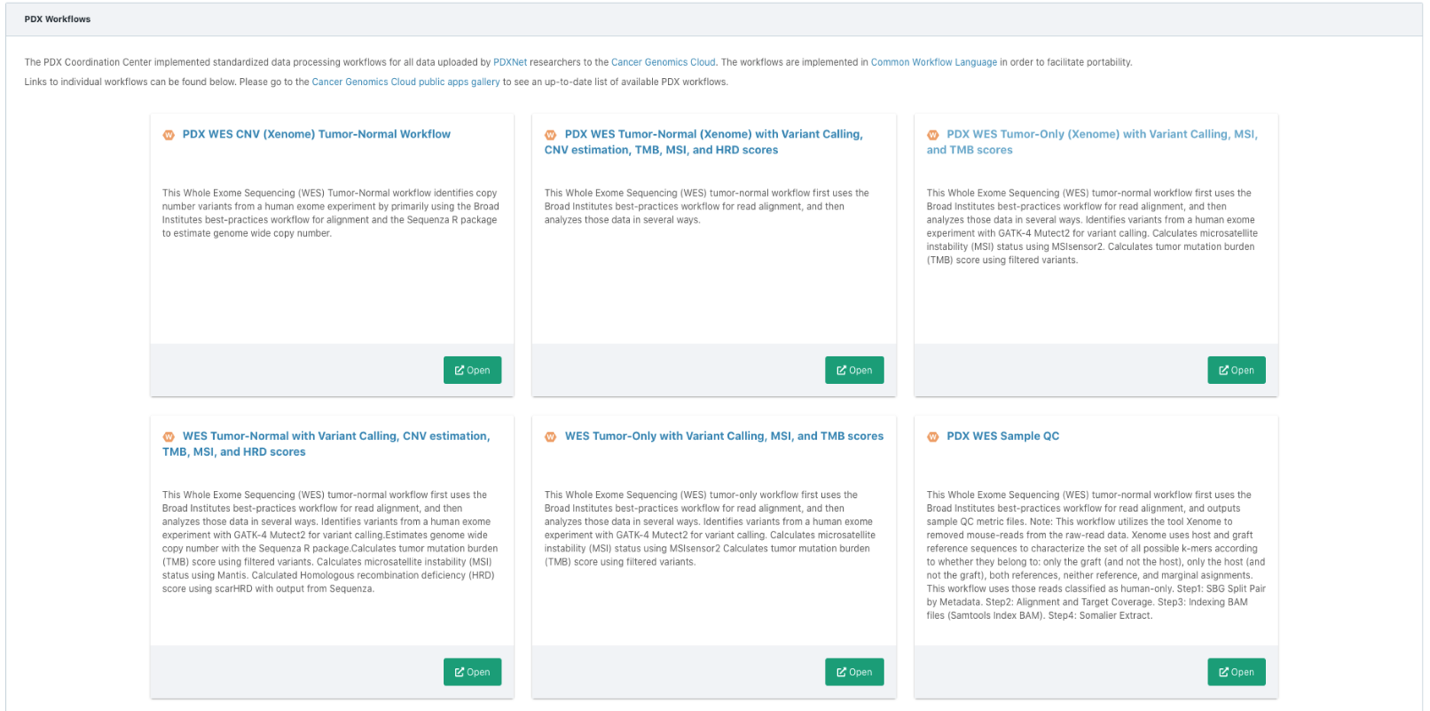


Supplement Figure 5. Standardized PDXNet processing workflows shown on the PDXNet Portal

Figure shows a section of the workflow page on the PDXNet portal. The page includes brief descriptions of standardized workflows created to process RNA-Seq, whole exome, and to a lesser extent array data. The page includes links to comprehensive workflow documentation on the Cancer Genomics Cloud Public Apps Gallery; where the workflows are made publicly available.


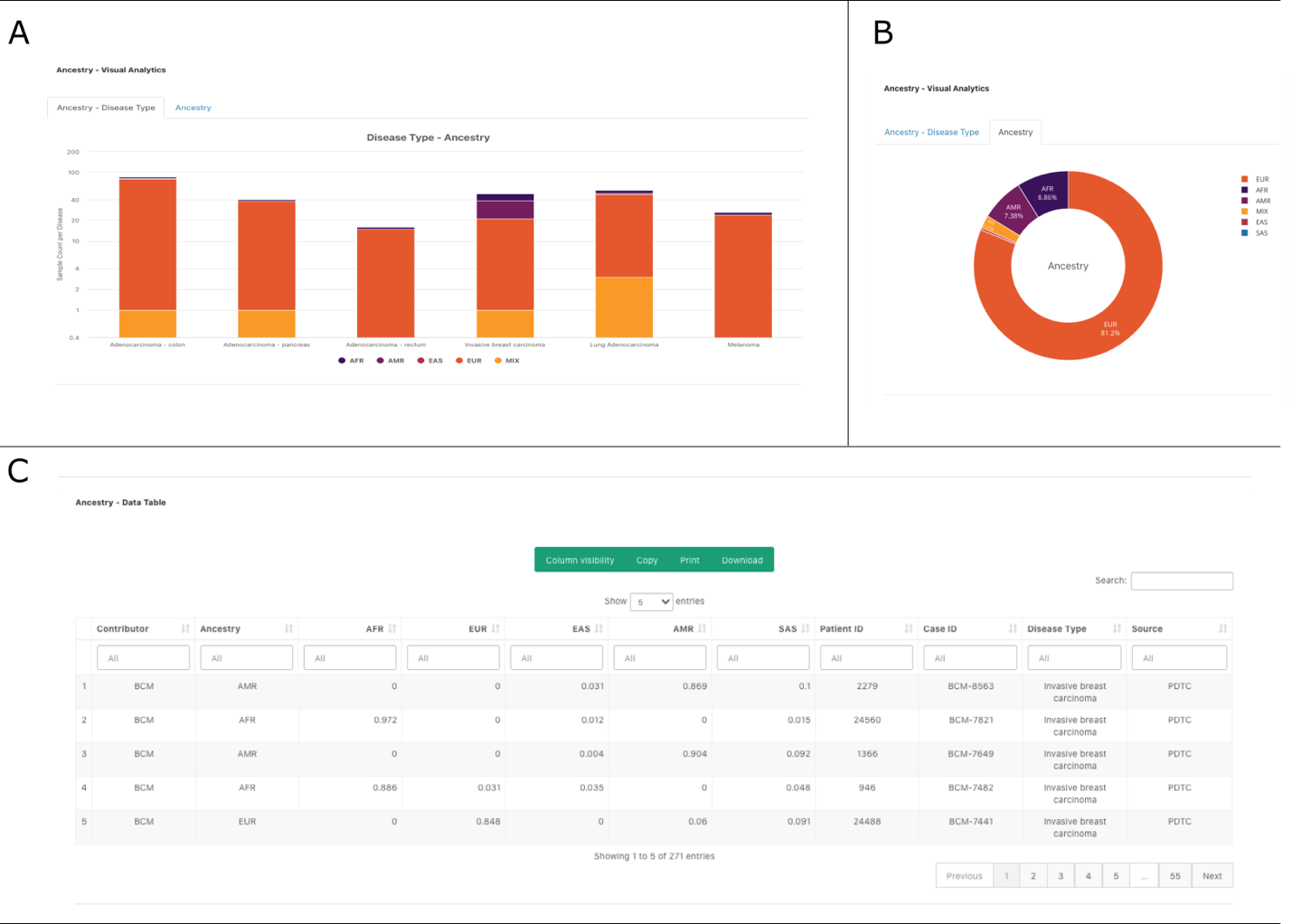


Supplement Figure 6. Ancestry information computed from sequencing data shown on the PDXNet Portal

Figure shows plots generated on the ancestry data page of the PDXNet portal **(A)** Panel A shows a stacked bar chart with each bar corresponding to a user selected disease. Each bar shows the ancestry composition of available samples by color. The ancestry algorithm classifies samples as African (AFR), American (AMR), East Asian (EAS), South Asian (SAS), and Mixed (MIX). **(B)** Panel shows computed ancestry in a pie chart. Ancestry is classified in the following categories: European(EUR), African(AFR), American(AMR), Mixed(MIX), East Asian(EAS), South Asian(SAS). **(C)** Panel shows ancestry metadata for the processed sequencing data in a spreadsheet format. The interface supports searching and sorting metadata. Users can copy, print, and download metadata into accessible formats.
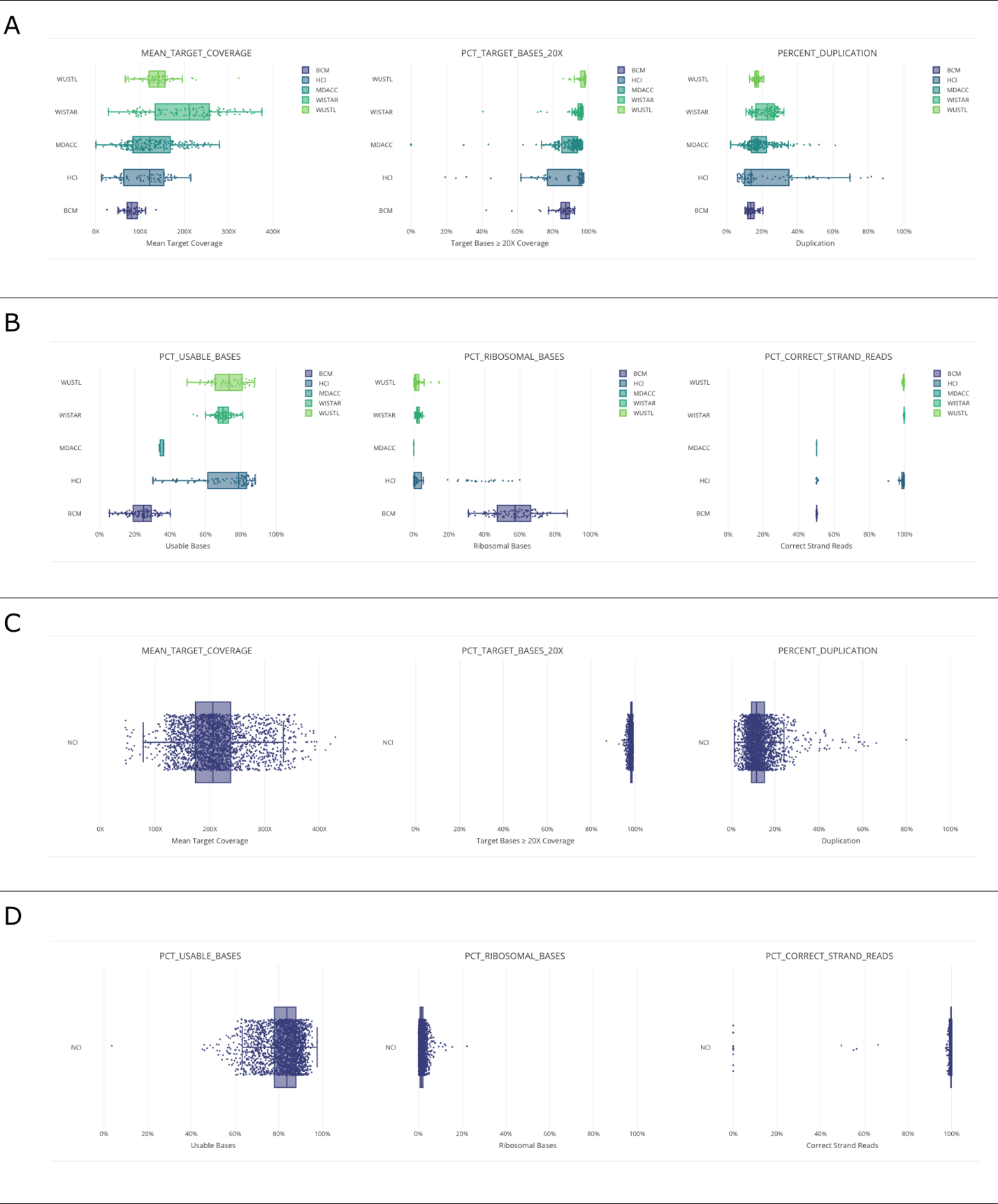


Supplement Figure 7. Sequencing quality control plots examples generated on the PDXNet Portal

Figure shows sequencing data QC figures generated on the quality control page of the PDXNet Portal (A) Plot shows mean target coverage, percent target coverage at 20x, and percent duplication as box plots with data for each PDX Development and Trial Center presented as a different box plot. (B) Plot shows percent usable basis, percent ribosomal basis, and percent correct strand reads as box plots with data for each PDX Development and Trial Center presented as a different box plot. (C) Plot shows mean target coverage, percent target coverage at 20x, and percent duplication as box plots generated from PDMR data. (D) Plot shows percent usable basis, percent ribosomal basis, and percent correct strand reads as box plots generated from PDMR data.
